# An efficient and secure technique for image steganography using a hash function

**DOI:** 10.7717/peerj-cs.1157

**Published:** 2022-11-24

**Authors:** Zahid Iqbal Nezami, Hamid Ali, Muhammad Asif, Hanan Aljuaid, Isma Hamid, Zulfiqar Ali

**Affiliations:** 1 Department of Computer Science, The Superior University Lahore, Lahore, Punjab, Pakistan; 2 Department of Computer Science, National Textile University, Faisalabad, Pakistan; 3 Computer Sciences Department, College of Computer and Information Sciences, Princess Nourah bint Abdulrahman University, Riyadh, Riyadh, Saudi Arabia; 4 Department Computer Science, National University of Technology, Islamabad, Pakistan

**Keywords:** Stegnopraphy, Hash function, LSB replacement, Data hiding, MSE, PSNR, Caesar cipher

## Abstract

Steganography is a technique in which a person hides information in digital media. The message sent by this technique is so secret that other people cannot even imagine the information’s existence. This article entails developing a mechanism for communicating one-on-one with individuals by concealing information from the rest of the group. Based on their availability, digital images are the most suited components for use as transmitters when compared to other objects available on the internet. The proposed technique encrypts a message within an image. There are several steganographic techniques for hiding hidden information in photographs, some of which are more difficult than others, and each has its strengths and weaknesses. The encryption mechanism employed may have different requirements depending on the application. For example, certain applications may require complete invisibility of the key information, while others may require the concealment of a larger secret message. In this research, we proposed a technique that converts plain text to ciphertext and encodes it in a picture using up to the four least significant bit (LSB) based on a hash function. The LSBs of the image pixel values are used to substitute pieces of text. Human eyes cannot predict the variation between the initial Image and the resulting image since only the LSBs are modified. The proposed technique is compared with state-of-the-art techniques. The results reveal that the proposed technique outperforms the existing techniques concerning security and efficiency with adequate MSE and PSNR.

## Introduction

Steganography conceals secret data in a non-secret file to avoid visual detection. In Islam et al. (2021), steganography conceals information that appears out of the ordinary in private or secret data. Because they relate to how secret material is kept, steganography for cryptology is commonly misconstrued. The difference between the two is that steganography involves sensitive information, but steganography does not appear to have any hidden information. When a group or individual feels that information is locked inside of them, they do not want to understand it Voleti et al. (2021). The term “steganography” is derived from the Greek word “steganos,” which means “hidden writing”. There are two elements to the term steganos: “secret” and “graphic,” which means “writing.” steganography, on the other hand, is the process of text or secret messages that can be hidden in other media assets, such as a picture, text, video, or music. The terms “steganography” and “cryptography” are frequently used interchangeably. Steganography conceals the message while watermarking certifies its integrity, and cryptography scrambles it. In Sasmal & Mula (2021) the case of steganography, the sender must select a suitable message carrier before beginning the concealment process. A reliable steganographic method must be chosen to encrypt real information effectively. The sender can then use any current communication mechanism to convey the secret message to the receiver. After receiving the message, the recipient must decode the hidden information using the appropriate extraction procedure. Various relevant steganographic techniques are utilized to achieve security depending on the carrier’s kind (Ali & Uddin, 2019). The steganography technologies created during the past three decades have been thoroughly examined in Pilania et al. (2021). These tools’ strengths, weaknesses, applicability, and opportunity for future study are all represented in the comparative analysis of these tools based on the given parameters. Academics and professionals alike have a great deal of acceptance for the OpenPuff steganography tool. To confirm and defend the performance of the OpenPuff tool, this study additionally analyses its performance on a few hitherto unknown parameters. In order to address some of the problems with steganography approaches, the Integer Wavelet Transform methodology and JPEG (Joint Photograph Expert Group) compression are suggested in Pilania et al. (2022). Due to their inherent qualities, video cover images and JPEG compression increase hiding capacity. The suggested method’s resilience and imperceptibility are enhanced by the usage of integer wavelet transform. Through the use of assessment measures including MSE, PSNR, SSIM, and correlation coefficient (CC). The imperceptibility of the suggested work is examined.

Image steganography: when an image is used as a cover object in steganography. In most of these strategies, image pixel intensities are employed to disguise the information.

Network protocol steganography: Steganography uses a network protocol as a cover object, such as TCP, UDP, ICMP, or IP. In this case, steganography may be accomplished by utilizing unused header bits of network protocols.

Video steganography, the carrier for secret information in video steganography is a video (combination of pictures). Discrete cosine transformations (DCT) are commonly used to obscure information in each movie’s pictures. Video steganography employs a variety of video formats, including H.264, Mp4, MPEG, AVI, and others.

Audio steganography is the use of audio as a carrier for concealing information. Voice over IP (VOIP) has become a highly essential medium due to its popularity. This steganography employs digital audio formats such as WAVE, MIDI, AVI, MPEG, and others.

In text steganography, strategies such as capital letters, white spaces, number of tabs, like Morse code, and others, are utilized to achieve information concealing text steganography.

The main objective of the proposed hash-based data embedding method is to improve the security of secret data in the given cover image with minimum alteration so that the quality of the cover image remains unnoticeable to the human visual system.

The rest of the article is organized as follows. Related work is presented in Section 2. Sections 3 and 4 presents the proposed approach, experimental description, and results. Section 5 concludes the article.

## Related Work

Because of developing technology and the complexity of software applications, data security, integrity, and availability are jeopardized; as a result, it is necessary to safeguard such systems and data. A novel steganography approach inside RGB shading space is suggested in Rahman et al. (2020), to offer increased security compared to previous systems. They use a different picture quality evaluation methodologies. Compared to previous methodologies, the results show greater strength, intangibility, and security, proving the exploratory work’s success. For PSNR correlation, the recommended technique had a 3.6701 per cent better average score. In Dhawan & Gupta (2021), the author proposes a steganography classification system based on technical and non-technical steganography and classification according to its domain. The quality of Stego pictures, payload capacity, mean square error, and structural similarity are all issues connected to steganography (SSIM), image fidelity (IF), normalized cross-correlation (NCC), and resilience are all important factors to consider. This work aims to investigate and compare several steganography algorithms using characteristics such as PSNR, MSE, and Robustness. In Sahu & Swain (2020), a study of LSB image steganographic recent advances were conducted to improve steganographic performance, such as resilience, integration capacity, and the ability to find secret knowledge. In Prashanti & Sandhyarani (2015), the authors propose two strategies; the first strategy involves using data or hidden messages in the Image, while the second involves inserting a secret grey image within another grey image. The four-state table, which generates pseudorandom numbers, is employed for these strategies. The data that has been concealed will be inserted. Because pseudorandom numbers in the table are included in the LSB location of the randomly picked Image, both techniques are more accurate. In Goel, Gupta & Kaushik (2014-2015), the researches focuses on a new way of embedding hidden messages into cover images utilizing the LSB method with various progressions and a homomorphic cryptographic approach. In Wade et al. (2019), mean square error (MSE), peak signal to noise ratio (PSNR), structure similiarity index (SSIM), CPU time, histograms and feature similarity index measurement (FSIM) was used to compare the stego picture to the cover image. Their research and experiments reveal that their proposed approach is faster and more efficient than traditional LSB methods. In  Baby et al. (2015), the DWT based steganography techniques was presented, which involves using DWT steganographic technology, in which numerous RGB pictures are blended into a single RGB image. The colours red, green, and blue, are used in this room. These three-colour spaces are used to conceal info that is not visible. This gadget produces consistent experimental results. The authors compared the picture quality of the stego with the original image coverage using the PSNR and SSIM indexes. The PSNR and SSIM index values of the suggested technique are both high. The authors discover that their experimental results outperform earlier methodologies, and that data compression has increased their capacity to incorporate data. As a result, overall security is strong, with fewer noticeable modifications to the stego picture. In Gouthamanaath & Kangaiammal (2018), the authors create a state-of-the-art strategy in binary picture steganography, to reduce texture distortion. The invariant texture patterns are initially derived from the binary picture using rotation, compliment, and mirroring in this steganography. They also presented a computation based on the recommended measurement that was applied. According to practical results, the suggested stenographic approach has high statistical safety, great picture quality, and integration power. In Nusrati, Hanani & Karimi (2015), the authors presented a new genetic algorithm-based steganography technique. Until the secret information is embedded, the gadget identifies the optimal spots in the cover picture to implant it. It adjusts the histogram by reducing the bit size. The grid is sliced using genetic algorithms. Multiple LSBs are the subject of the next block notice. After the algorithm discovers the correct places, hidden blocks are inserted, and the main file is formed. Experimental findings imply that the high-quality stego method outperforms the LSB algorithm’s basic answer of the question, to find appropriate places in carrier image to embed the message with the least changes of bits. The improvement strategy was presented in Qazanfari & Safabakhsh (2014), to improve the LSB++, which causes the pixels to be covered against additional bits, resulting in less distortion of the co-occurrence matrices. The DCT coefficients in JPEG are retained. When using this method, fewer traces are required than when using the LSB+ method. Because the cover photo and stego image histograms are similar, this technique is shielded from histogram attacks. Stego images are of high quality since additional bits are not used. In Almazaydeh (2020), the authors used RGB image in steganography to improve data transfer security over the internet. The cover picture is a 24-bit RGB image with hidden details. In many bins, the X-Box mapping has 16 different values. “X” represents any integer from 0 to 9. The binary image’s LSBs are converted to X-Boxes. Since mapping is employed, retrieving the concealed details is difficult. As a result, it has strong digital defiance standards. PSNR also helps the stego picture’s quality. In Sai Charan et al. (2015), the authors transform ordinary text into ciphertext, which is then embedded in a colour image. The data is encrypted in two stages: the first stage employs the Caesar cipher technique, while the second stage employs chaos theory. The ciphertext is injected using a 3, 3, 2 LSB replacement approach after encryption. In Almawgani et al. (2022), a hybrid steganography approach is developed that uses the Haar Discrete Wavelet Transform (HDWT), the Lempel Ziv Welch (LZW) algorithm, the Genetic Algorithm (GA), and the Optimal Pixel Adjustment Process (OPAP). The proposed article separates the cover picture into (n x n) pixels in non-overlapping chunks. HDWT is used to improve the stego image’s resistance to assaults. The LZW algorithm is used for the secret message to boost the capacity and security of the concealed image. The secret message cover image coefficients are encoded and compressed using GA. The OPAP is used to lower the mistake rate.

## Proposed Approach

In the proposed approach, we use two things. Message encryption method and embedded algorithm

### Message encryption technique

Many encryption techniques can be used to protect data *i.e.*, symmetric-key cryptography and asymmetric-key cryptography. In the proposed technique, we use asymmetric-key cryptography to encrypt the secret message. The detail of asymmetric-key cryptography is as follows:

**Asymmetric-key cryptography:** Uses two keys, one to encrypt data and one to decrypt it. This type of cryptography is more secure than symmetric-key cryptography because it is harder for someone to intercept the decryption key. It is used in applications such as online shopping and file sharing.

#### Caesar cipher

The Caesar cipher encryption method is a type of symmetric-key cryptography that uses a substitution cipher. It was developed by Julius Caesar in the 1st century BC and is still used today. The Caesar cipher is named after its inventor, and it works by substituting random letters for the letters in a text message, intending to make it difficult to decipher. This type of encryption is used to protect sensitive information, such as passwords or financial data, and is considered one of the most secure methods available. In this method, certain places in the alphabet shift every letter inside a message. So, let us say shift 3, then the letter b will become e.

1. Take the secret message
2. Apply the encryption algorithm (Caesar cipher)
3. Save the cipher message

#### Vigenère cipher

The Vigenere cipher is a polyalphabetic substitution cipher in which each letter of the alphabet is replaced with a different letter according to a table of key values. The cipher was named after Francesco Vigenère, who published an algorithm for it in 1694. Polyalphabetic ciphers are useful for encrypting text that will be read by individuals other than the sender and receiver, as they are not as susceptible to cryptanalysis as monoalphabetic ciphers. The Vigenere cipher is based on a simple substitution algorithm: each letter of the alphabet is replaced with one of its 26 corresponding letters. To encrypt a message using this cipher, a person would start by selecting a key value (26 total), determining which letters will be used to replace the corresponding letters in your message. Table 1 lists the key values and corresponding letters for English alphabets.

**Table 1:** Key values and corresponding letters for English alphabets for Vigenere cipher.

	A	B	C	D	E	F	G	H	I	J	K	L	M	N	O	P	Q	R	S	T	U	V	W	X	Y	Z
A	A	B	C	D	E	F	G	H	I	J	K	L	M	N	O	P	Q	R	S	T	U	V	W	X	Y	Z
B	B	C	D	E	F	G	H	I	J	K	L	M	N	O	P	Q	R	S	T	U	V	W	X	Y	Z	A
C	C	D	E	F	G	H	I	J	K	L	M	N	O	P	Q	R	S	T	U	V	W	X	Y	Z	A	B
D	D	E	F	G	H	I	J	K	L	M	N	O	P	Q	R	S	T	U	V	W	X	Y	Z	A	B	C
E	E	F	G	H	I	J	K	L	M	N	O	P	Q	R	S	T	U	V	W	X	Y	Z	A	B	C	D
F	F	G	H	I	J	K	L	M	N	O	P	Q	R	S	T	U	V	W	X	Y	Z	A	B	C	D	E
G	G	H	I	J	K	L	M	N	O	P	Q	R	S	T	U	V	W	X	Y	Z	A	B	C	D	E	F
H	H	I	J	K	L	M	N	O	P	Q	R	S	T	U	V	W	X	Y	Z	A	B	C	D	E	F	G
I	I	J	K	L	M	N	O	P	Q	R	S	T	U	V	W	X	Y	Z	A	B	C	D	E	F	G	H
J	J	K	L	M	N	O	P	Q	R	S	T	U	V	W	X	Y	Z	A	B	C	D	E	F	G	H	I
K	K	L	M	N	O	P	Q	R	S	T	U	V	W	X	Y	Z	A	B	C	D	E	F	G	H	I	J
L	L	M	N	O	P	Q	R	S	T	U	V	W	X	Y	Z	A	B	C	D	E	F	G	H	I	J	K
M	M	N	O	P	Q	R	S	T	U	V	W	X	Y	Z	A	B	C	D	E	F	G	H	I	J	K	L
N	N	O	P	Q	R	S	T	U	V	W	X	Y	Z	A	B	C	D	E	F	G	H	I	J	K	L	M
O	O	P	Q	R	S	T	U	V	W	X	Y	Z	A	B	C	D	E	F	G	H	I	J	K	L	M	N
P	P	Q	R	S	T	U	V	W	X	Y	Z	A	B	C	D	E	F	G	H	I	J	K	L	M	N	O
Q	Q	R	S	T	U	V	W	X	Y	Z	A	B	C	D	E	F	G	H	I	J	K	L	M	N	O	P
R	R	S	T	U	V	W	X	Y	Z	A	B	C	D	E	F	G	H	I	J	K	L	M	N	O	P	Q
S	S	T	U	V	W	X	Y	Z	A	B	C	D	E	F	G	H	I	J	K	L	M	N	O	P	Q	R
T	T	U	V	W	X	Y	Z	A	B	C	D	E	F	G	H	I	J	K	L	M	N	O	P	Q	R	S
U	U	V	W	X	Y	Z	A	B	C	D	E	F	G	H	I	J	K	L	M	N	O	P	Q	R	S	T
V	V	W	X	Y	Z	A	B	C	D	E	F	G	H	I	J	K	L	M	N	O	P	Q	R	S	T	U
W	W	X	Y	Z	A	B	C	D	E	F	G	H	I	J	K	L	M	N	O	P	Q	R	S	T	U	V
X	X	Y	Z	A	B	C	D	E	F	G	H	I	J	K	L	M	N	O	P	Q	R	S	T	U	V	W
Y	Y	Z	A	B	C	D	E	F	G	H	I	J	K	L	M	N	O	P	Q	R	S	T	U	V	W	X
Z	Z	A	B	C	D	E	F	G	H	I	J	K	L	M	N	O	P	Q	R	S	T	U	V	W	X	Y

English alphabet key values A ->Z

Latin alphabet key values 1 2 3 4 5 6 7 8 9 10 11 12 13 14 15

### Hash function

Hash functions are mathematical algorithms that create a unique identifier for any item. A hash function takes an input and produces a fixed-length output. The output of a hash function is unique for every item it is used on. This act makes hash functions an excellent way to create a unique identifier for an item. The hash algorithm used to create the identifier can also be used to verify the integrity of the data. This property is useful for database lookup, file identification, and password hashing applications.

This study used the hash function to secure the message. The hash function takes the number of a pixel as an input and returns a number based on a key value. The key value is already exchanged between the sender and receiver. We elaborate on this with an example:

Suppose we want to hide a bit in pixel 30 of the cover image, and the key is 13 (The sender and receiver can use any value as a key), then the hash function returns the value 4. We again apply a hash function using 4 (this shows that we want to hide the message in one of the 4 LSBs), which returns a ‘zero’. We add ‘1’ in this value and hide the message bit at first LSB.

The above example shows that as we change the key value (instead of 13), the bit of the cover image will be changed to hide the message bit of the same pixel 30. So, in this case, no one can find the message bit because the third party does not know the exact bit of the cover image, which hides the message bit.

**Figure 1:**
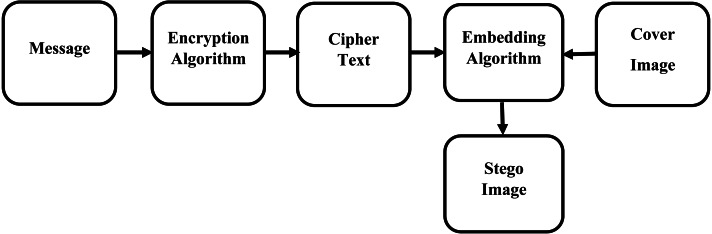
Encoding flow diagram.

### Embedding algorithm

**Figure 2:**
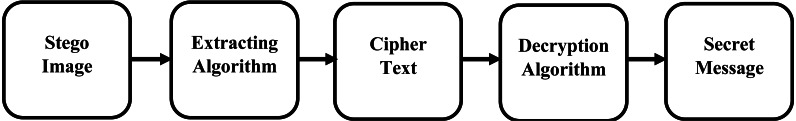
Decoding flow diagram.

The embedded and extracted algorithm works on both sides, sender and receiver, as shown in Figs. 1 and 2 respectively.

### Sender side

Algorithm 1 is used on the sender’s side; this algorithm hides the encrypted cipher text message within the cover image.

**Table:** 

**ALGORITHM 1: The embedded algorithm on the sender side**
***Input:*** *As input, this function will take the encrypted text message prepared in section III-A and the Cover Image.*
**Output:** *It will print the Stego Image that has been encoded.*
***Begin***
1 *Read the Cover Image and cipher Text Message.*
2 *Use the Encryption Technique to secure the message, we can use (1) Caesar Cipher or (2) Vigenère cipher*
3 *Traverse through the Image*
4 ***fori****= 1 to****height***
5 ***for j****= 1 to****width***
6 ***If****more bits are remaining to embed*
7 *Find the Least Significant Bit of the current pixel using a hash function*
8 ***SB1****= mod (((****i***−1) * ***height****+****j****), 13)*
9 ***SB****= mod (****SB1****,4)*
10 *Replace the message bit with Least Significant bit****SB***
11 *Update the current pixel of output with a new value*
12 *if the pixel value increases, replace all the right-side bits of the changed bit with zeros. and if the pixel value decreases, then replace the right bits of the changed bit with ones.*
13 ***End***
14 **End**
15 ***End***
16 *Calculate the****MSE****using the input and output pixel values*
17 *Calculate the****PSNR****using the calculated****MSE***
18 *Save the Stego image*
19 **End**

### Receiver end

Algorithm 2 is used on the receiver side; this algorithm extracts the encrypted cipher text message within the cover image.

**Table:** 

**ALGORITHM 2: The extracted algorithm on the receiver side**
***Input:*** *The input to this function will be a Stego Image.*
**Output:** *The decrypted Target Text Message from Stego Image will be produced.*
***Begin***
1 *Find the text message size****S****and set*S1= S * 8, i.e., the bitstream size.
2 Take a blank bit string named extracted bits, such as **M**
3 *Traverse through the Stego image*
4 ***fori****= 1 to****height***
5 ***for j****= 1 to****width***
6 ***If****more message bits are remaining to be extracted*
7 *Find the Least Significant Bit of the current pixel using the hash function used on the sender side*
8 ***SB1****= mod (((****i***−1) * ***height****+****j****), 13)*
9 ***SB****= mod (****SB1****,4)*
10 *Store the LSB of the pixel in bit string M*
11 ***End***
12 **End**
13 ***End***
14 *Get all the bits in 8 column table where each row is the bits of the character in the hidden text*
15 *Convert the extracted bits to characters by multiplying with powers of 2*
16 *Apply Caesar cipher or Vigenère cipher which one used on the sender side*
17 *Print the hidden text*
18 **End**

## Experimental Description and Results

**Figure 3:**
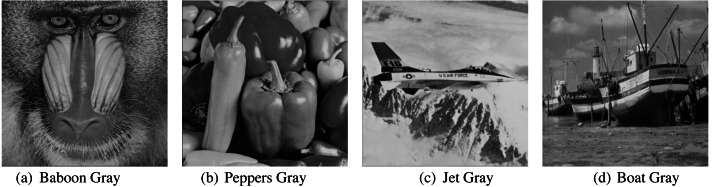
Cover images.

In this article, we used MATLAB to carry out the research. We implemented the proposed strategy in MATLAB 2018. A machine with an Intel(R) Core (TM) i7-7500U CPU running at 2.70 GHz, 2.90 GHz and 8 GB of RAM is used for the tests. We used 10 to encrypt the message using cipher technique (we can use any number instead of 10 for encryption purpose). We also use 13 as a hash function in this study but we can use any number instead of 13. Four typical grayscale images were used as cover images to test the suggested technique. These images are called ‘Baboon’, ‘Boat’, ‘Jet’, and ‘Pepper’. They all have a resolution of 512×512 pixels, as seen in Fig. 3.

### Qualitative analysis

#### Histogram analysis

**Figure 4:**
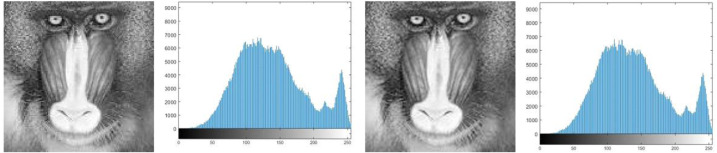
(A) Cover image of baboon, (B) histogram of baboon cover image, (C) baboon stego image, (D) histogram of baboon stego image.

**Figure 5:**
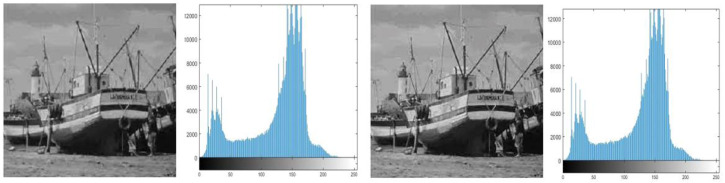
(A) Cover image of boat, (B) histogram of boat cover image, (C) stego image of boat, (D) histogram of boat stego image.

**Figure 6:**
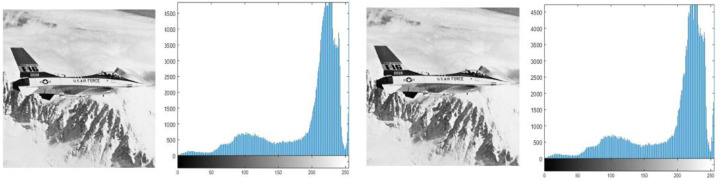
(A) Cover image of jet, (B) histogram of jet cover image, (C) stego image of jet, (D) histogram of jet stego image.

**Figure 7:**
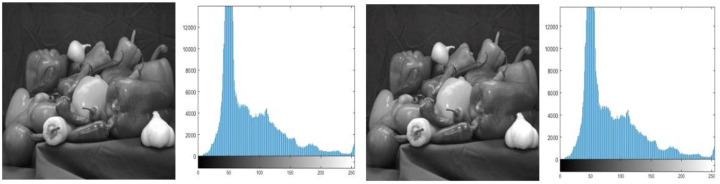
(A) Cover image of peppers, (B) histogram of preppers cover image, (C) stego image of peppers, (D) histogram of peppers stego image.

The goal of histogram analysis is to figure out how the stego image compares to the cover image. The stego image’s imperceptibility was demonstrated via histogram analysis. The histograms of the cover and stego images are compared in Figs. 3, 4, 5, 6 and 7. The results of this study reveal minor changes in the histograms of both images, which means the difference between the stego, and cover images is not visible.

### Quantitative analysis

#### Compared with state of the arts techniques

Based on the following assessment parameters: payload, bits per pixel (bpp), PSNR, and mean square error, the results of the proposed approach are compared with those of existing techniques already in use  (Hamza et al., 2021). Tables 2, 3, 4 and 5 presents the photos’ comparison, including the Baboon, Jet, Boat, and Peppers. In the proposed technique, it is hard to find the bit used to save the message compared to many other existing techniques. The bit pattern can be easily predicted, so the proposed technique is more secure than other techniques with minimal MSE.

**Table 2:** Gray stego image of baboon.

**Author**	**Bits per pixels (bpp)**	**Payload**	**MSE**	**PSNR**
Shehzad & Daǧ (2019)	0.25	65,536	0.12	57.19
Liu, Chang & Chien (2017)	1	262,144	0.39	52.17
Muhammad et al. (2015)	0.08	21,845	0.42	51.89
Setiadi & Jumanto (2018)	1.2	314,572	1.43	46.57
Kuo, Kuo & Wuu (2015)	0.8	221,987	2.24	44.62
Islam et al. (2016)	1.56	410,636	2.4	44.37
Celik et al. (2002)	0.26	74600	7.71	39.26
Yang, Sun & Sun (2009)	2.9	785572	7.89	39.16
Mohamed & Mohamed (2016a)	3.1	838,860	8.4	38.85
Jung & Yoo (2015)	2.97	778,567	10.66	37.85
Khalind & Aziz (2013)	2.25	589,824	11.45	37.54
Xuan et al. (2002)	0.32	85507	14.22	36.6
Shehzad & Dag (2017)	2,00	524,288	21.1	34.92
Muhammad et al. (2017)	0.33	87,381	23.88	34.35
Hamza et al. (2021)	3.01	789,270	26.98	33.82
Chikouche & Chikouche (2017)	0.003	1,024	65.02	30
Hong, Chang & Li (2020)	1	262,144	130.34	26.98
Proposed technique	1	262,144	2.65	43.89

**Table 3:** Gray stego image of jet.

**Author**	**Bits per pixels (bpp)**	**Payload**	**MSE**	**PSNR**
Liu, Chang & Chien (2017)	1	262,144	0.39	52.17
Muhammad et al. (2015)	0.08	21845	0.41	51.92
Setiadi & Jumanto (2018)	1.1	288,358	1.07	47.82
Kuo, Kuo & Wuu (2015)	0.9	238872	1.31	46.93
Swain (2016)	3.1	814497	3.64	42.51
Jung & Yoo (2015)	2.97	778,567	9.95	38.15
Hussain et al. (2018)	3.15	824756	10.81	37.79
Khalind & Aziz (2013)	2.25	589824	11.09	37.68
Muhammad et al. (2017)	0.33	87381	16.71	35.9
Bai et al. (2017)	3.19	1,024,983	26.85	33.84
Hamza et al. (2021)	3.58	939,592	26.85	33.84
Tseng & Leng (2014)	3.15	825753	28.38	33.6
Hong, Chang & Li (2020)	1	262,144	41.31	31.97
Proposed technique	1	262,144	2.61	43.95

**Table 4:** Gray stego image of boat.

**Author**	**Bits Per Pixels (bpp)**	**Payload**	**MSE**	**PSNR**
Liu, Chang & Chien (2017)	1	262,144	0.39	52.12
Muhammad et al. (2015)	1.1	288,358	0.91	48.51
Kuo, Kuo & Wuu (2015)	0.84	222,589	2.13	44.84
Zakaria et al. (2018)	3.28	833,482	3.78	42.35
Yang, Sun & Sun (2009)	2.87	753832	7.81	39.2
Jung & Yoo (2015)	2.97	778,567	10.3	38.38
Hussain et al. (2018)	3.09	810,735	11.24	37.62
Khalind & Aziz (2013)	2.25	589,824	11.61	37.48
Swain (2016)	3.22	846,516	14.03	36.66
Bai et al. (2017)	0.33	87,381	17.66	35.66
Hamza et al. (2021)	3.27	858,580	28.91	33.52
Tseng & Leng (2014)	3.1	812,646	36.81	32.47
Hong, Chang & Li (2020)	1	262,144	41.31	31.97
Proposed technique	1	262,144	2.63	43.92

**Table 5:** Gray stego image of peppers.

**Author**	**Bits Per Pixels (bpp)**	**Payload**	**MSE**	**PSNR**
Liu, Chang & Chien (2017)	1	262,144	0.39	52.12
Muhammad et al. (2015)	0.08	21,845	0.41	51.99
Setiadi & Jumanto (2018)	1.09	285,736	0.97	48.24
Kuo, Kuo & Wuu (2015)	0.8	222,054	2.27	44.57
Mohamed & Mohamed (2016b)	2..25	589,824	2.36	44.4
Yang, Sun & Sun (2009)	2.99	786,016	8.07	39.06
Hussain et al. (2018)	3.09	810,501	9.05	38.56
Swain (2016)	3.11	815,912	9.61	38.33
Liao, Wen & Zhang (2011)	3.07	805,492	10.81	39.79
Jung & Yoo (2015)	2.97	778,567	10.89	38.08
Khalind & Aziz (2013)	2.25	589,824	11.56	37.5
Bai et al. (2017)	3.05	810,024	12.24	37.25
Khodaei, Sadeghi Bigham & Faez (2016)	3.13	822,042	12.59	37.13
Muhammad et al. (2017)	0.33	87,381	21.73	34.76
Tseng & Leng (2014)	3.16	828,375	28.31	33.61
Hamza et al. (2021)	3.26	854,796	29.44	33.44
Hong, Chang & Li (2020)	1	262,144	29.58	33.42
Proposed technique	1	262,144	2.9	43.49

Table 2 compares the suggested method to known LSB steganography methods for the stego picture of a baboon. This comparison demonstrates that the suggested approach embeds 262,144 secret data bits with 43.89 PSNR and 2.65 MSE in 262,144 pixels of the baboon cover picture.

Table 2 shows that the proposed technique is secure and outperforms 11 out of 17 exiting techniques in MSE when using a baboon as a cover image. Compared to the other two techniques having the same payload, the proposed technique performs better than one existing technique.

Table 3 compares the suggested method to current LSB steganography methods for the Jet stego picture. This comparison demonstrates that the suggested method embeds 262,144 secret data bits with 43.95 PSNR and 2.61 MSE in 262,144 pixels of the Jet cover image. Table 3 shows that the proposed technique is secure and outperforms 9 out of 13 exiting techniques in MSE when using a Jet as a cover image. Compared to the other two techniques having the same payload, the proposed technique performs better than one existing technique.

Table 4 compares the suggested method to current LSB steganography methods for the Boat stego picture. This comparison demonstrates that the suggested method embeds 262,144 secret data bits with 43.92 PSNR and 2.63 MSE in 262,144 pixels of the Boat cover image. Table 4 shows that the proposed technique is secure and outperforms 10 of 13 exiting techniques in terms of MSE when using a Boat as a cover image. Compared to the other two techniques having the same payload, the proposed technique performs better than one existing technique.

Table 5 compares the suggested and current LSB steganography methods for the Peppers stego picture. This comparison demonstrates that the suggested method embeds 262,144 secret data bits with 43.49 PSNR and 2.90 MSE in 262,144 pixels of the Peppers cover Image. Table 5 shows that the proposed technique is secure and outperforms 12 out of 17 exiting techniques in MSE when using Peppers as a cover image. Compared to the other two techniques having the same payload, the proposed technique performs better than one existing technique.

## Conclusion & Future Scope

This study demonstrates a very secure LSB image steganography algorithm. The proposed technique used the Caesar cipher and Vigenere to encrypt the message and the hash function for steganography. The suggested algorithm employs a cover image in the spatial domain for secret hiding information. The proposed technique improves security and speed compared to the standard LSB steganography approaches. In the future, we will increase the payload and add a security method to hide the secret message in each cover image.

##  Supplemental Information

10.7717/peerj-cs.1157/supp-1 Supplemental Information 1 Code

10.7717/peerj-cs.1157/supp-2 Supplemental Information 2 DataSet used in the project

## Additional Information and Declarations

### Data Availability

The following information was supplied regarding data availability: The data and code are available in the Supplemental Files. The data was taken from ResearchGate: https://www.researchgate.net/figure/Ten-original-grayscale-test-images-a-Airplane-b-Baboon-c-Boat-d-Couple-e-Elaine-f_fig5_353280794.
